# Adjustable Trajectory Design Based on Node Density for Mobile Sink in WSNs

**DOI:** 10.3390/s16122091

**Published:** 2016-12-09

**Authors:** Guisong Yang, Shuai Liu, Xingyu He, Naixue Xiong, Chunxue Wu

**Affiliations:** 1 Department of Computer Science and Engineering, School of Optical-Electrical and Computer Engineering, University of Shanghai for Science and Technology, Shanghai 200093, China; gsyang@usst.edu.cn (G.Y.); shuailiu@st.usst.edu.cn (S.L.); wcx@usst.edu.cn (C.W.); 2 Shanghai Key Lab of Modern Optical Systems, Shanghai 20093, China; 3 Public Experiment Center, University of Shanghai for Science and Technology, Shanghai 200093, China; 4 College of Electronic and Information Engineering, Tongji University, Shanghai 201804, China

**Keywords:** movement trajectory, mobile sink, node density, Hilbert space-filling curve, power control

## Abstract

The design of movement trajectories for mobile sink plays an important role in data gathering for Wireless Sensor Networks (WSNs), as it affects the network coverage, and packet delivery ratio, as well as the network lifetime. In some scenarios, the whole network can be divided into subareas where the nodes are randomly deployed. The node densities of these subareas are quite different, which may result in a decreased packet delivery ratio and network lifetime if the movement trajectory of the mobile sink cannot adapt to these differences. To address these problems, we propose an adjustable trajectory design method based on node density for mobile sink in WSNs. The movement trajectory of the mobile sink in each subarea follows the Hilbert space-filling curve. Firstly, the trajectory is constructed based on network size. Secondly, the adjustable trajectory is established based on node density in specific subareas. Finally, the trajectories in each subarea are combined to acquire the whole network’s movement trajectory for the mobile sink. In addition, an adaptable power control scheme is designed to adjust nodes’ transmitting range dynamically according to the movement trajectory of the mobile sink in each subarea. The simulation results demonstrate that the proposed trajectories can adapt to network changes flexibly, thus outperform both in packet delivery ratio and in energy consumption the trajectories designed only based on the network size and the whole network node density.

## 1. Introduction

With the fast spread of communication technology and Internet of Things applications, wireless sensor networks (WSNs) are being widely used in many applications such as medical care, military surveillance and environmental monitoring [1,2], where they have become an important and effective means of information gathering in various fields.

One main goal of WSNs is to gather the sensing data and send them to the sink. In traditional WSNs, a static sink is used to gather the sensing data. However, with the limited energy and communication range in a large scale sensor network, it is not practical for all nodes to send the sensing data to the sink directly. Hence, the static sink concept has some defects like poor network connectivity and low packet delivery ratio, as well as high energy consumption as a result of unbalanced flow distribution. More seriously, it also incurs in the hot-spot problem [3], which will cause network partition that further degrades the network lifetime.

To address the above problems, designing the predefined trajectory that the mobile sink can move along to collect sensing data [4,5,6,7,8,9,10] has attracted the extensive attention of many researchers. By fully exploiting the predefined mobility, the network coverage is improved and the number of transmissions is decreased to some extent. However, in this case, the mobile sink may not visit all the areas frequently, which will inevitably cause packet loss and high end-to-end delay. Therefore how to cover the whole network and gather the data efficiently becomes a big challenge.

To cope with this challenge, Viana focused on the space-filling curve [11]. Space-filling curves have the property that they ensure the filling of a d-dimension space by traversing every pre-defined point in a region once and only once, in a specific order. Furthermore, Ghafoor proposed a mobile sink trajectory design method based on the Hilbert space-filling curve [12], in which the trajectory was designed by analyzing the network size. To further deal with networks with high node density, Ghafoor improved the previous trajectory by taking the node density of whole network into consideration [13]. However, in networks with non-uniform or uneven node deployment, this method would result in a significant decrease in packet delivery ratio for high re-transmission rates.

To address this problem, we propose a path-constrained mobile sink trajectory design method, in which the trajectory is designed based on the node density in each subarea. To ensure the full coverage of the network and efficient data gathering, the Hilbert space-filling curve [14] is employed and the mobile sink moves along it. In addition, the proposed movement trajectory of the mobile sink can adapt to networks with different node densities.

The trajectory is designed as follows: firstly, by analyzing of the maximum communication range of the sensor nodes and network size *L*, the order *k* of the Hilbert curves can be calculated to ensure full coverage of the network, and then the network is divided into 4*^k^* subareas. Secondly, by calculating a proper Hilbert curve order in each subarea, the trajectory in each subarea is determined based on the node density. Thirdly, the trajectory is formed by combining the above Hilbert curves from all subareas. For example, by analyzing the node density in a subarea, the proper order *d_i_* of the Hilbert curve in this subarea can be calculated and form the corresponding Hilbert curve as the movement trajectory in it. After acquiring the orders of the Hilbert curve in all subareas and getting their corresponding Hilbert curves, all these trajectories are combined into a continuous filling curve, which is the final movement trajectory for the mobile sink, so in essence, the mobile sink can adjust its movement trajectory based on the node density in each subarea so as to adapt to the network status.

In addition, we also propose an adaptable power control scheme. In each subarea, the transmission range of sensor nodes can be adjusted in order to increase the energy efficiency, thus increasing the network lifetime. The main contributions of the work are as follows:

1. We design an adjustable trajectory (that follows the Hilbert curve) based on the node density in each subarea for mobile sink;
2. We propose an adaptable power control scheme to adjust nodes’ transmitting range in each subarea according to the order of Hilbert curve in it in order to reduce the energy consumption.

Comprehensive simulations are conducted with different node densities or different kinds of node deployments to determine the performance of the proposed method both in terms of packet delivery ratio and in average energy consumption, which is compared with classic methods such as the network size-based and the whole network node density-based strategy.

The rest of the paper is organized as follows: in Section 2, related works are presented. In Section 3, the system model is introduced. Section 4 gives the detailed design of the proposed method. The performance evaluation of the proposed method is presented in Section 5, and finally we conclude the paper in Section 6.

## 2. Related Work

In traditional WSNs, all the sensor nodes as well as the sink node are static, which would result in the hot-spot problem [3]. To address this problem, many research works are drawn on leveraging the mobility of the sink node to make the energy be consumed evenly. The first thought is to make the sink move according to a predefined path. Many studies have been done using mobile sinks with predefined paths [4,5,6,7,8,9,10]. In [4], the mobility pattern of a path-constrained mobile sink was analyzed. The results showed that the path-constrained approaches can reduce the network overhead. In [5], the authors explained the advantage of path-constrained sink mobility, and analyzed the performance of five different mobile sink deployment methods. In [6], the authors proposed a data gathering scheme using path-constrained mobile sinks, in which the network was divided into two parts according to the communication range of the mobile sinks and the distance to the sensor nodes to increase the flexibility. In [7], a one-hop data gathering scheme was proposed, in which a three-layer network structure was used to increase the scalability and network lifetime. In [8], a low-complexity data gathering protocol with one or more mobile sinks was proposed to reduce the routing overhead and prolong the network lifetime. In [9], by resolving the Geometric Sink Trajectory problem, the authors proposed a trajectory planning method for multiple mobile sinks, in which the packet delay and the network lifetime were balanced. In [10], the authors proposed a predefined trajectory for multiple mobile sinks in a network divided into hexagonal tiles, in which the trajectory is designed to make the mobile sink act as the bottleneck node in the network and thus reduce the risk of energy holes.

These approaches can all reduce the energy consumption of the sensor nodes, thus prolonging the network lifetime, but they are not flexible enough to deal with the issues of complicated networks, for example, to address the problem that a movement trajectory has to cover the whole network so as to improve the packet delivery ratio in the network. Therefore the space-filling curve concept was introduced to make the data gathering more efficient.

In [11], the authors first introduced the notion of space-filling curves to WSNs and proposed the opportunistic delivery algorithm to increase the packet delivery ratio. In [12], a sink trajectory design which is based on the Hilbert space-filling curve was proposed by Ghafoor for full coverage of the network and less delivery delay, but in a network with high node density, this trajectory would lead to poor delivery ratios and high average delivery delays. To solve this problem, Ghafoor also proposed a novel method [13] which improves upon the previous one by taking the node density of the whole network into consideration. By considering both the network size and node density, the efficiency of data gathering is ensured, while the network coverage is guaranteed. However, Ghafoor only considered the node density of the whole network other than that of a specific subarea, that is, in a large scale sensor network that can be divided into many subareas where the nodes are randomly deployed, the node density of subareas may be non-uniform or uneven, and when the node density in some subareas is relatively high, while in rest of the subareas it is relatively low, the above method can hardly adapt to this situation, which it can lead to a decrease of the packet delivery ratio and an increased energy consumption.

To further increase the flexibility of the network, another kind of approaches using random or controllable trajectories were proposed [15,16,17,18,19]. In [15], the authors proposed a high-reliability data gathering protocol based on mobile sinks, which adopts the concept of state machine to achieve efficient routing and reduce the overhead. In [16], a sink tracking method was proposed, in which the nodes track the mobile sink by receiving the broadcast message sent by a mobile sink periodically. In order to further reduce the overhead, a sink tracking method called DAMST [17] was also proposed, in which energy efficiency was high while the overhead is cut down significantly. In [18], a continuous and optimal trajectory was proposed, in which the concept of support vector regression is used to determine the optimal trajectory of a mobile sink to maximize the network lifetime in event-driven sensor networks. In [19], the authors proposed a Greedy Scanning Data Collection Strategy (GSDCS), in which the network is divided into many grids and the sensor nodes in the same grid form a cluster to increase the efficiency of data gathering. In each cluster, a cluster head is selected based on the residual energy of each node as well as the distance between each node and the center point of the corresponding grid cell to collect the sensing data. The trajectory of the mobile sink is dynamically adjusted according to the network status by using a scanning trajectory. Finally, by tracking the location of the mobile sinks, the sensing data are relayed to the mobile sinks by the cluster heads to prolong the network lifetime. However, for a network where the mobile sinks can move randomly or uncontrollably, the sensing data has to be sent to it by tracking its real time location, and the tracking of the mobile sinks brings extra overhead in computation and storage. In our work, the movement trajectories are predefined, and the only additional overhead is introduced by the power control messages, which can be ignored compared with the location updating message overhead.

## 3. System Model

In many real applications, sensor nodes are deployed unevenly or non-uniformly due to an unfavorable geographical location. In order to make the movement trajectory of the mobile sink adapt to networks with uneven node deployment or different node densities, the network needs to be partitioned into many subareas, so we can design an adjustable movement trajectory based on the node density in each specific subarea. Therefore, the network needs to be partitioned first according to the network size to ensure the full coverage of the network.

### 3.1. Network Model

In this paper, we introduce the following assumptions: sensor nodes with limited initial energy *E* are deployed in a square of size *L*, the communication range of sensor nodes is adjustable with a maximum value *r_max_*. The network is divided into some subareas according to network size, and each subarea is a square of size *l*, the total number of subareas is *N*. A mobile sink used to gather the sensing data periodically starts from the bottom left of the network and travels at a constant speed *v* following a predefined movement trajectory. For the mobile sink, when gathering data in a subarea, each sensor node only sends one packet with a fixed size to it directly.

### 3.2. Hilbert Space-Filling Curve

In this paper, to address the problem of full coverage of network, the trajectory is designed based on a space-filling curve. By using a space-filling curve, the mobile sink can traverse every defined point in the network once and only once, thus all the sensing data can be gathered in a timely and efficient way.

**Figure 1:**
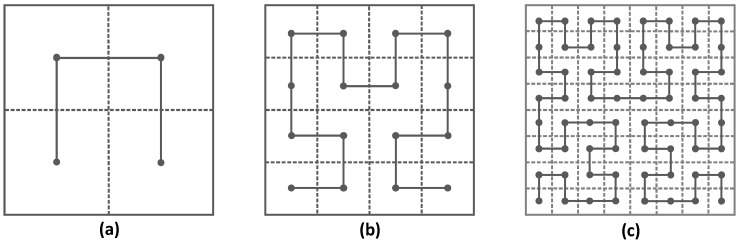
The first three orders of Hilbert space-filling curve: (**a**) 1-order (*k* = 1, *N* = 4); (**b**) 2-order (*k* = 2, *N* = 16); (**c**) 3-order (*k* = 3, *N* = 64).

The Hilbert space-filling curve [9] is a space-filling curve proposed by David Hilbert in 1891. Hilbert curves can map a one-dimensional line *l* onto a two-dimensional square *S*. For instance, if we map a line *l* onto square *S*, firstly line *l* is split into four identical sub-intervals and the square *S* into four identical sub-squares, where each sub-interval can be continuously mapped onto one sub-square. If this continues, *l* and *S* are partitioned into 4*^k^* identical duplications for *k* = 1, 2, 3, where *k* defines the order of the Hilbert curve. Finally the curves traverses over 4*^k^* sub-squares in two dimensions. The correspondence between the line intervals and the sub-squares is shown in Figure 1, in which the first, second and third order of Hilbert curve are depicted.

The points on the curve show the correspondence between sub-squares and sub-intervals, so adjacent sub-intervals always correspond to adjacent sub-squares. The transformation of a curve of order *k* − 1 into a curve of order *k* can be viewed as a replacement of each sub-square of the order *k* − 1 with a 1-order curve.

Besides Hilbert curves, there are many other space-filling curves such as the Z curve, Grey-coded curve, etc. [20]. In [21], the authors compared the above space-filling curves and found that the Hilbert curve outperforms the others in the locality preservation property that can increase the data gathering efficiency, which is the main reason Hilbert curve is adopted as the mobile sink movement trajectory in this paper.

### 3.3. Hilbert Curves with Heterogeneous Orders

After partitioning according to network size, the network can be divided into many subareas. Here, the number of subareas is defined by the order in which the mobile sink traverses them. Recalling that the total number of subareas is *N*, the subarea that the mobile sink traverses first is called C_1_ while the subarea the mobile sink traverses last is called C_N_. In this work, the mobile sink moves into an area from the bottom left and exits from the bottom right.

**Figure 2:**
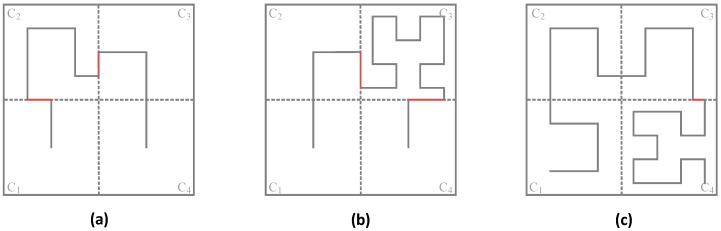
Three different kinds of Hilbert curves with heterogeneous orders: (**a**) 1-order curve in C_1_, C_3_, C_4_ and 2-order curve in C_2_; (**b**) 1-order in C_1_, C_2_, C_4_ and 3-order curve in C_3_; (**c**) 2-order curve in C_1_, C_2_, C_3_ and 3-order in C_4_.

Figure 2a shows Hilbert curves with heterogeneous orders, where the Hilbert curve with 1-order in C_1_, C_3_ and C_4_ while the Hilbert curve with 2-order in C_2_, for the subarea C_2_ has relatively high node density, C_2_ so adopts the 2-order Hilbert curve while others adopt 1-order Hilbert curve. Similarly, Figure 2b,c show Hilbert curves with heterogeneous orders in different subareas according to the node density in them.

It should be noted that, when the orders of Hilbert curves in two adjacent subareas are different from each other, there exists a “gap” at the border of the two adjacent subareas (the red line in Figure 2), and so the movement trajectory becomes discontinuous. To get a continuous movement trajectory, the “gap” on the border needs to be connected by using the red lines as shown in Figure 2.

### 3.4. Energy Model

Considering the communication range is relatively short, this paper adopted the free-space channel model [22]. Let $\varepsilon_{fs}$ be the energy require by the amplifier in free-space channel, $\alpha_{tx}$ and $\alpha_{rx}$ be the energy consumed during transmitting and receiving per bit respectively.

The energy consumed during the node *i* transmits β bit data to node *j* is described as follows:

(1) $$E_{tx}(i,j)=(\alpha_{tx}+\varepsilon_{fs}D_{\left(i,j\right)}^{2})\beta ,$$

where $D_{\left(i,j\right)}$ is the distance between node *i* and node *j*.

The energy consumed by node *j* that receives β bit data is described as follows:

(2) $$E_{rx}(j)=\alpha_{rx}\beta ,$$

The total energy consumed by node *i* and node *j* during the above two processes can be defined as follows:

(3) $$E_{total}(i,j)=(\alpha_{tx}+\alpha_{rx}+\varepsilon_{fs}D_{\left(i,j\right)}^{2})\beta ,$$

## 4. Adjustable Trajectory Design Based on Node Density

The design of the adjustable trajectory for mobile sink consists of three main steps. In the first step, to ensure the full coverage of the network, we should get the proper order of the Hilbert curve (that is *k*) according to the network size. Then, to prepare for the second step, the whole network is divided into 4*^k^* subareas. Once the subareas are determined, they will remain unchanged. In the second step, to adapt to networks with different node densities, especially the highly dense networks with non-uniform or uneven node deployment, we design the trajectory in each subarea by analyzing the node density in it. To distinguish from the order *k* of the Hilbert curve according to network size, the order of the Hilbert curve used in subarea C_i_ based on node density is denoted by *d_i_*. Finally in the third step, the adjustable trajectory based on node density for the mobile sink can be acquired by combining the trajectories in all subareas and the trajectories on all borders. In addition, in order to relieve the network congestion and prolong the network lifetime, we propose an adaptable power control scheme according to the characteristics of the adjustable trajectory, which can adjust the transmission power of sensor nodes in specific subareas.

### 4.1. The Order of the Hilbert Curve According to the Network Size

In the first step of our method, to guarantee the full coverage of the network, the proper order of the Hilbert curve should be selected according to the network size. The proper order here means the minimum order Hilbert curve that can fill the full network.

**Figure 3:**
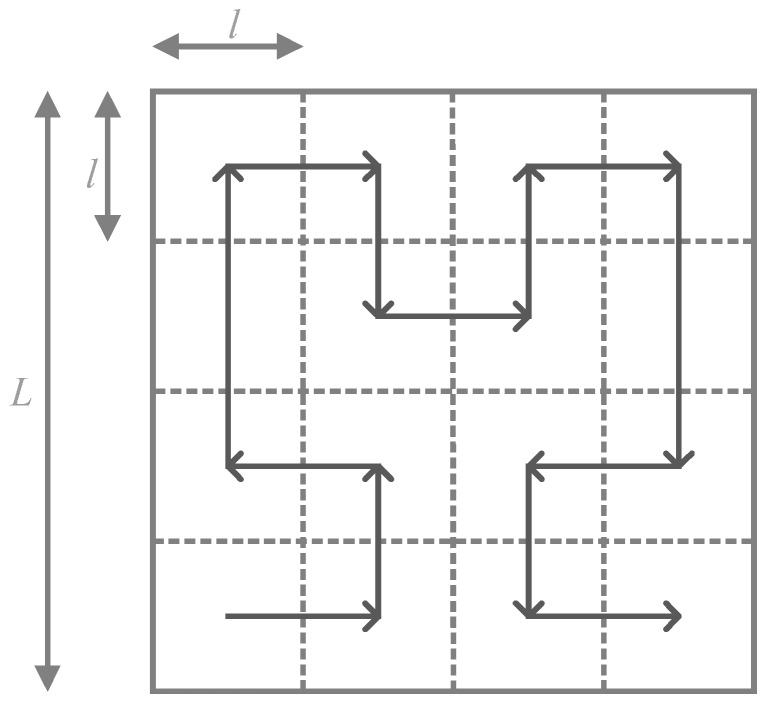
The length of the network and subarea.

Recall that the network is a square area of size *L*, and suppose that the network can be divided into *N* subareas and the subareas are smaller sub-squares of size *l*, as shown in Figure 3. The following Equations (4)–(8) calculating order *k* of the Hilbert curve are cited from [11].

The *l* can be defined by *L* and *N* as follows:

(4) $$l=\frac{L}{\sqrt{N}},$$

To ensure the sensor node in each subarea can communicate with the mobile sink directly, the inequality between the size of a subarea *l* and the maximum communication range of sensor node *r_max_* holds:

(5) $$l\leq \frac{1}{\sqrt{2}}r_{\max},$$

Supposed that the proper order of the Hilbert curve is *k*, the network can be divided into $N=4^{k}$ subareas, and then the size of a subarea *l* can be defined as:

(6) $$l=\frac{L}{2^{k}},$$

By combing Equations (5) and (6), we have:

(7) $$\min2^{k}\geq \frac{\sqrt{2}L}{r_{\max}},$$

After some manipulation, *k* can be obtained as follows:

(8) $$k=\left\lceil \log_{2}\left\{\frac{\sqrt{2}L}{r_{\max}}\right\}\right\rceil ,$$

Through the above calculation, the minimum order *k* of the Hilbert curve which can fill the whole network can be obtained. Through Equation (8), it can be known that the value of *k* depends on the network size *L* and the sensor nodes’ maximum communication range *r_max_*. When *k* = 2, the trajectory of the mobile sink is as shown in Figure 3.

It is clear that, if the movement trajectory for the mobile sink is designed only according to the network size when using Hilbert space-filling curves, and since the mobile sink travels at a constant speed, the time it stays in each subarea must be a fixed value. In a highly dense network, the mobile sink can hardly collect sensing data completely within a limited traversing time due to the communication congestion, and thereby this results in a decrease of the packet delivery ratio and the network lifetime.

### 4.2. The Order of the Hilbert Curve Based on Node Density in Each Subarea

In Section 4.1, the minimum order *k* of the Hilbert curve which can fill the whole network is obtained according to the network size. However, in a highly dense network with uneven node deployment, this kind of trajectory according to the network size may hardly complete data gathering within a limited traversing time, and this will result in a significant decrease in packet delivery ratio.

In the second step of our method, to further improve the trajectory design according to network size, the difference of node density in each subarea is taken into consideration to make the trajectory adaptable to networks with uneven node deployment in high node density situations. For this we can design an adaptable trajectory based on node densities in specific subareas.

After getting the proper order *k* of the Hilbert curve according to the network size, the whole network can be divided into *N* = 4*^k^* subareas. In an adjustable trajectory design based on node density, the node density in each subarea is adopted to calculate a proper Hilbert curve order in this subarea. And hence, the movement trajectories for the mobile sink of the whole network are acquired by combining all the trajectories in these subareas.

After acquiring the curve order *k* of the Hilbert curve according to the network size, it can be seen that in each subarea (except in the first and last subarea), the length of the curve in this subarea is the same as its side length. Therefore, the traversing time of the mobile sink in subarea C_i_ can be calculated as follows:

(9) $$t_{i}=\frac{l_{i}}{v},$$

where *l_i_* stands for the side length of subarea C_i_ and *v* stands for the travel speed of the mobile sink.

Since each sensor node only sends one data packet to the mobile sink during the process of data gathering, thus in subarea C_i_, we define *n_i_max_* as the maximum number of sensor nodes that the mobile sink can communicate with during its traversing time, then *n_i_max_* can be calculated from Equation (10), where *t_avg_* represents the average time for transmitting the sensing data:

(10) $$n_{i\_\max}=\frac{t_{i}}{t_{avg}},$$

Subarea C_i_ has its own Hilbert curve order based on its node density, and the curve order in all subareas are initialized with *k*, so a curve order that is not less than *k* will guarantee the full coverage of the network. Here *n_i_max_* is used as the threshold to decide whether the mobile sink can complete data gathering in its traversing time in C_i_ or not.

In a network with unevenly deployed nodes, if the number of nodes *n_i_* in C_i_ obeys *n_i_* < *n_i_max_*, the order *d_i_* of the Hilbert curve equals the initialized order *k* of the Hilbert curve according to the network size, which means that the mobile sink can complete data gathering during its traversing time in this subarea. Otherwise, if the number of nodes *n_i_* in C_i_ obeys *n_i_* ≥ *n_i_max_*, we would try to use *k* + 1 as the curve order in C_i_ first, and consequently C_i_ is further divided into four smaller sub-squares, and the new traversing time *t_i_new_* of the mobile sink in subarea C_i_ is extended to:

(11) ti_new=4×(12liv)=2ti,

Since the average transmission time for a data packet *t_avg_* is a fixed value and the new traversing time *t_i_new_* the mobile sink takes becomes twice *t_i_*, hence the maximum number of sensor nodes the mobile sink can gather is also doubled.

Based on the analysis above, the proper traversing time of the mobile sink in subarea C_i_ can be acquired by using Equation (11). Therefore, the order *d_i_* of the Hilbert curve based on node density in C_i_ can be calculated from the proper traversing time of the mobile sink in C_i_ as follows:

(12) $$\left\{ \begin{array}{l} d_{i}=k,n_{i}<n_{i\_\max}\\ d_{i}=k+\left\lceil \log_{2}\left(\frac{n_{i}}{n_{i\_\max}}\right)\right\rceil ,n_{i}\geq n_{i\_\max} \end{array}\right.,$$

**Figure 4:**
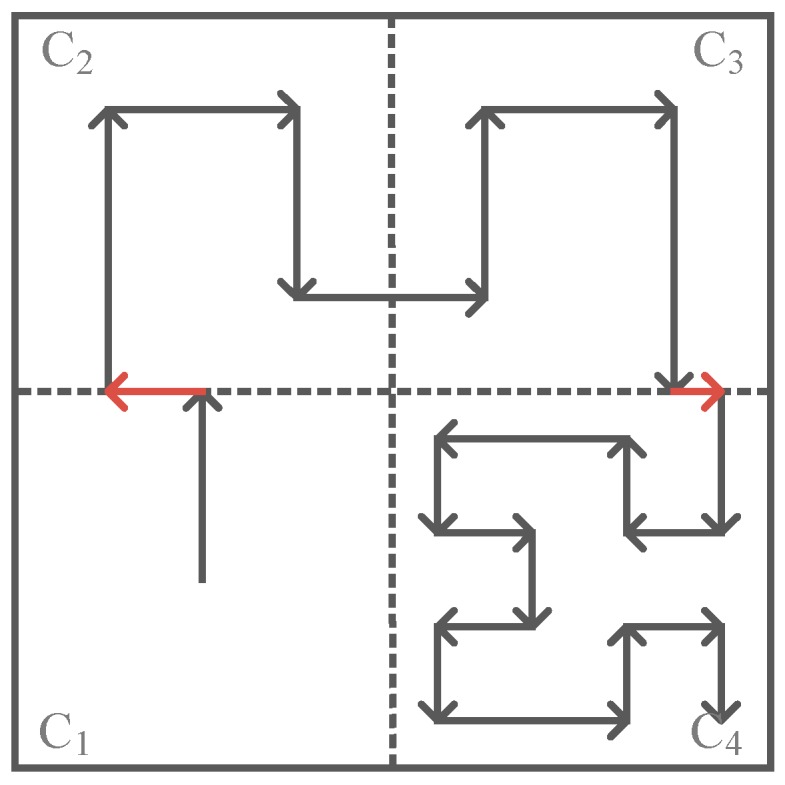
An example of an adjustable trajectory based on node density in specific subareas.

Finally, the trajectories in all subareas are combined to form the final movement trajectory for the mobile sink. The adjustable trajectory based on node density in this network is shown in Figure 4. In this case, suppose that the network is divided into four subareas (C_1_ to C_4_) according to its network size. The node density in C_1_ is the lowest one where the mobile sink moves following a 1-order Hilbert curve; the node density in C_2_ and C_3_ is higher than that in C_1_ where the mobile sink moves following a 2-order Hilbert curve; and the node density in C_4_ is the highest one, where the mobile sink moves following a 3-order Hilbert curve.

Algorithm 1 gives the detailed calculation of the order of the Hilbert curve based on node density in a specific subarea.

**Algorithm 1.** The Order of the Hilbert Curve Based on Node Density in a Specific Subarea1. **Input**
*L*, *r_max_*, *v*, *t_avg_*;2. **Output**
*d_i_* (*i* = 1, 2, 3, …, *N*.);3. Initialize the variables (e.g., *k*←0, *N*←0);4. Calculate the order *k* of the Hilbert curve according to Equation (8);5. Calculate the number of the subareas *N*;6. ***for*** each subarea *C_i_*: *i* = 1, 2, …, *N*.7.  Calculate the node density *n_i_*;           *//n_i_ is the number of nodes in C_i_*8.  Calculate the traversing time *t_i_* of the mobile sink according to Equation (9);9.  Calculate the threshold *n_i_max_* according to Equation (10);10.  Calculate the order *d_i_* of the Hilbert curve according to Equation (12);11. ***End for***

It should be noted that using a Hilbert curve with heterogeneous orders would result in a discontinuous trajectory. If the two adjacent subareas adopt different curve orders, there exists a “gap” on the border of the two adjacent subareas, and so the movement trajectory become discontinuous since the exit location of previous subarea is not the entrance location of next subarea. To keep the trajectory continuous, we should design the movement trajectory for the mobile sink on the border of two adjacent subareas.

### 4.3. Trajectory Design on Borders

**Figure 5:**
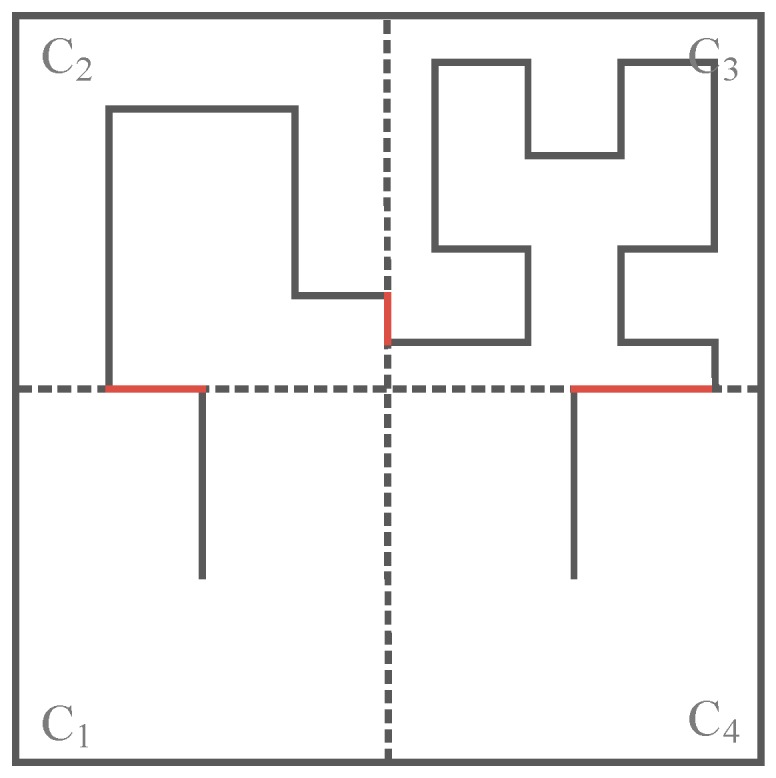
Two adjacent subareas with different curve orders will have a “gap” on their border (the red line).

The trajectory design on borders will be discussed in the third step of our method in this section. It can be known from Section 4.2, that for an adjustable trajectory design based on node density in specific subareas, the order of the Hilbert curve in each subarea may be different. Consequently, as shown in Figure 5, there will be a “gap” (the red line in Figure 5) on the trajectory in the border of two adjacent subareas with different curve orders. The border here means the common edge of two adjacent subareas.

To combine these curves and make the whole trajectory a continuous curve, we then discuss how to design the trajectory on the border of two adjacent subareas in the following paragraphs. When the order *k* of the Hilbert curve is acquired according to the network size in the first step of our method, the number of subareas *N* is also determined. Thus the number of trajectories on borders that need to be designed is *N* − 1.

To deal with the trajectory design on borders, the key is to get the length of the trajectory on the border and the traversing direction of the mobile sink on the border trajectory, respectively.

#### 4.3.1. The Length of the Trajectory on the Border

To get the length of the trajectory on a border, we should firstly determine the order *d_i_* of the Hilbert curve in subarea C_i_ and the order *d_i+_*_1_ of the Hilbert curve in subarea C_i+1_, and fill the specific subarea using the Hilbert curve, then the exit location of previous subarea C_i_ and the entrance location of next subarea C_i+1_ can be obtained. Once obtained the specific exit and entrance locations on the border of the two adjacent subareas, the length of the trajectory on border can be calculated as follows:

(13) $$l_{border\_trajectory}=\left|\frac{L}{2^{d_{i}+1}}-\frac{L}{2^{d_{i+1}+1}}\right|,$$

where *d_i_*, *d_i+_*_1_ stand for curve orders based on the node density in C_i_, C_i+1_, respectively.

#### 4.3.2. The Traversing Direction of a Mobile Sink on the Border Trajectory

The current traversing direction of the mobile sink is set as a reference direction. The traversing direction we need to design for mobile sink on border trajectory is the direction relative to the reference direction. Considering that when the mobile sink leaves one subarea, its traversing direction is perpendicular to the border, the traversing direction of the mobile sink on the border trajectory could only be one of two directions: left or right.

The traversing direction of the mobile sink on the border trajectory must fulfill the following rules: in the case of a curve order increase, which means that *d_i+_*_1_ > *d_i_*, if the current traversing direction of the mobile sink is vertical to the border, the traversing direction for the mobile sink on the border is left, and if the current traversing direction of the mobile sink is horizontal to the border, the traversing direction for the mobile sink on the border is right; in the case of curve order reduction, which means that *d_i+_*_1_ < *d_i_*, the traversing direction we need to design is the opposite of the direction designed in the case of curve order increase.

**Figure 6:**
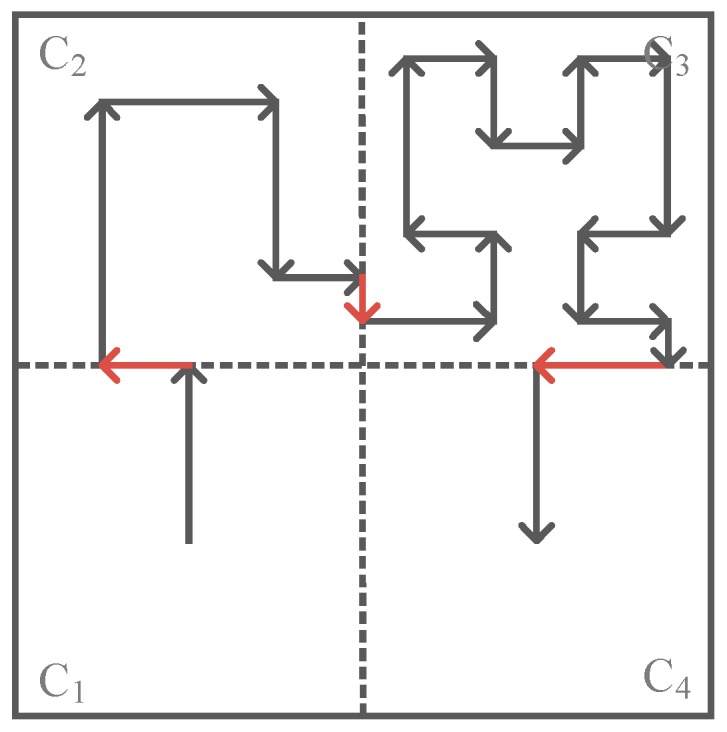
The traversing trajectory design on border (the red line).

Figure 6 shows a network divided into four subareas according to its network size, where C_1_ and C_4_ adopt 1-order Hilbert curves, C_2_ adopts a 2-order Hilbert curve and C_3_ adopts a 3-order Hilbert curve according to their node density.

Recalling that if two adjacent subareas have different curve orders, there will be a “gap” on the border of them, in this case, since C_1_ adopts a 1-order Hilbert curve while C_2_ adopts a 2-order Hilbert curve, the exit location of the trajectory in C_1_ does not meet the entrance location of the trajectory in C_2_, thus the trajectory becomes discontinuous, and accordingly the border trajectory (red line between C_1_ and C_2_ in Figure 6) is employed to connect the discontinuous trajectory. Similarly, the “gaps” between C_2_ and C_3_, C_3_ and C_4_ are also filled with a border trajectory.

It is worth noticing that the traversing direction of the mobile sink on the border trajectory is associated with the following two factors: current traversing direction of the mobile sink and whether the curve order is increased or reduced between the two adjacent subareas.

The detailed design of the border trajectory is shown in Algorithm 2.

**Algorithm 2.** The Detailed Design of the Trajectory on a Border1. **Input**
*d_i_*, *d_i+_*_1_, *cur_dir*;     //*cur_dir* is the current traveling direction of mobile sink2. Calculate the length of the trajectory on border *l_border_trajectory_* according to Equation (12);3. ***if*** (*d_i_*! = *d_i+_*_1_)            *//when curve order d_i_ ≠ d_i+_*_1_4.   ***if*** (*d_i_* < *d_i+_*_1_)            *//curve order is increased*5.   ***if*** (*cur_dir* = left or *cur_dir* = right)6.                   *//current traversing direction of mobile sink is horizontal*7.    *direction_on_border*←right; 8.   ***else***9.    *direction_on_border*←left;10.    ***end if***11.   Mobile sink traverses along the direction of *direction_on_border* with the length *l_border_trajectory_*;12.   ***else***               *//curve order is decreased*13.   ***if*** (*cur_dir* = up or *cur_dir* = down)14.                   *//current traversing direction of mobile sink is vertical*15.     *direction_on_border*←left;16.   ***else***17.    *direction_on_border*←right;18.    ***end if***19.   Mobile sink traverses along the direction of *direction_on_border* with the length *l_border_trajectory_*;20.   ***end if***21. ***end if***

In Algorithm 2, to determine whether to employ the border trajectory, the two curve order *d_i_*, *d_i+_*_1_ in C_i_, C_i+1_ are compared firstly. When *d_i_* = *d_i+_*_1_, the trajectory in C_i_ and C_i+1_ can connect to each other directly and there is no necessity to employ the border trajectory. Otherwise the border trajectory is employed according to the method presented above. Finally the mobile sink can move following the border trajectory on the border when there is a “gap” between two adjacent subareas, thus the entire traversing trajectory becomes a continuous curve.

### 4.4. Adaptable Power Control

We recall that in Section 4.1, in a given network size *L*, the value of *k* is determined, the curve order *k* and the number of subarea *N* holds *N* = 4*^k^*, and so the number of subareas is also a determined value. In this case, when the mobile sink moves into a subarea, nodes in the subarea can communicate with it using a constant transmission range. In Section 4.2, the curve order *d* is selected based on the node density in each subarea, so in some highly dense subareas, a relatively high order of the Hilbert curves will be selected.

Since the mobile sink will traverse all the subareas to collect the sensing data in them, therefore, in some subareas that nodes are densely deployed, the mobile sink moves following the high order of the Hilbert curves. It takes more time to traverse these subareas, which can decrease the distance between a sensor node and the mobile sink, and accordingly, we can use power control to adjust the transmission range the sensor node uses to communicate with the mobile sink in such a subarea, which can effectively reduce the energy consumption, so it can be deduced that in the subareas where nodes are densely deployed, the higher the curve order *d*, the shorter the transmission range a node needs to communicate with the mobile sink, and accordingly, the energy consumed can also be reduced.

After the first step of our method, assuming that node *a* in subarea C_1_ will transmit data to the mobile sink, the distance between node a and the mobile sink is *l*_1_. Here we note that C_1_ is a subarea where nodes are densely deployed, so a curve order *d*_1_ that is larger than *k* is selected based on the node density in C_1_ to design the movement trajectory of the mobile sink, and in this case, the distance between node *a* and the mobile sink becomes *l*_2_, and *l*_2_ holds *l*_1_ > *l*_2_.

In free-space channel, the energy saved for node a transmitting β bits data to the mobile sink can be defined as:

(14) $$E_{save}=\left(l_{1}^{2}-l_{2}^{2}\right)\varepsilon_{fs}\beta ,$$

Therefore, it can be seen that the energy consumption of the sensor is reduced by using power control to adjust its transmission range.

**Figure 7:**
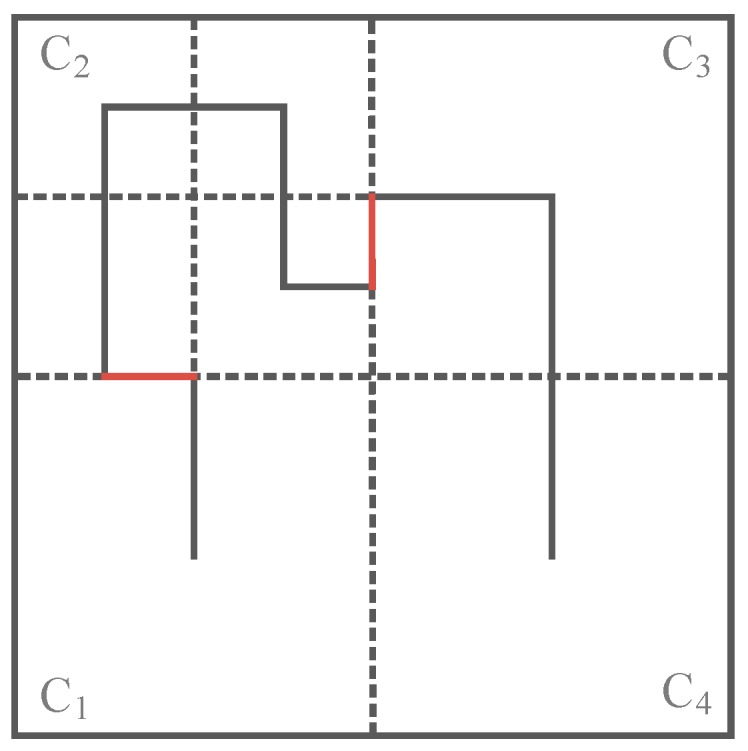
An example of adjustable power control.

For example, in Figure 7, the mobile sink follows a 2-order Hilbert curve in subarea C_2_ where nodes are densely deployed, and we can use power control to adjust the transmitting range the sensor node used to communicate with the mobile sink, so the transmission range of the sensor node in C_2_ can be reduced from $\frac{\sqrt{2}L}{2}$ to $\frac{\sqrt{2}L}{4}$ according to Equation (5). Thus sensor nodes in C_2_ can adjust their transmission range in order to reduce the energy consumption when transmitting their sensing data to the mobile sink.

To enable sensor nodes in such subareas to adjust their transmission range to communicate with the mobile sink in a timely fashion, the mobile sink periodically broadcasts a power control message to sensor nodes as it moves into a subarea. The format of the power control message is as shown in Table 1, which includes the message type, target subarea ID and the new transmission range.

The “Message type” field indicates the type of the message, which is mainly used to differentiate the power control messages from the other messages used in network. “Target subarea ID” field is used to indicate which subarea this message is supposed to be sent to. The “New TX range” field is used to set the new transmission range for the sensor nodes in the target subarea.

The new transmission range of the sensor node *r_TX_i_* is determined by the network size *L* and the curve order *d_i_*, and can be defined as follows:

(15) $$r_{TX\_i}=\frac{\sqrt{2}L}{2^{d_{i}}},$$

Each sensor node has its own subarea ID. On receiving the power control message, a node will decide whether to accept it or not by comparing the “Target subarea ID” in the message with its own subarea ID. Since each subarea may adopt different Hilbert curve orders and thus the minimum required transmitting range of sensor nodes differs, the power control message has to indicate the target subarea in case the message was received by nodes in a wrong subarea. If the “Target subarea ID” equals the nodes’ own subarea ID, a node then updates its transmission range using the value in “New TX range”, otherwise, it just ignores this message. Once the transmission range is updated, the sensor node can communicate with the mobile sink by using it in this subarea.

## 5. Performance Evaluation

Our simulations are performed under OMNeT++ 4.6 with the INET framework [23]. The parameters used in this simulation are listed in Table 2.

By following the Hilbert curve to design the movement trajectory of the mobile sink, in this simulation, we compare our proposed adjustable trajectory based on node density in specific subareas with the trajectory based on network size [12], and the trajectory based on node density of the whole network [13], to evaluate its efficiency. In this section these three kinds of trajectories are denoted as ad-based, k-based and d-based trajectories, respectively.

In this work, we mainly concentrate on the comparing parameters such as the packet delivery ratio (PDR) and the energy consumption. In this simulation, sensor nodes are deployed in an 800 × 800 m^2^ network. Two kinds of communication models which are the single-hop model and cluster model are adopted to test the performance of the proposed method. The reason we choose these two kinds of communication models is that they can fit the requirements in different applications.

In Table 2, the transmission power of the sensor node ranges from 0.05 mW to 2.6 mW. In fact, the transmission power also limits the communication range of the sensor nodes. In the cluster model, the minimum transmission power is used in the intra-cluster communication and the adaptive power control method can adjust the transmission power of the cluster head (CH). In this scheme, the CH is selected randomly and each node can and only can belong to one CH at a time. In the process of data gathering, the sensor nodes send their sensing data to the CHs first and then the CHs relay these sensing data to the mobile sink as the mobile sink passes by.

We set six scenarios with different node deployments. In Scenarios 1 and 3, sensor nodes are deployed uniformly, while in Scenarios 2, 4, 5 and 6, the sensor nodes are deployed unevenly. Then the mobile sink can move following the trajectory to collect the sensing data.

### 5.1. Evaluation of the Packet Delivery Ratio in a Single-Hop Model

**Scenario 1.** Sensor Nodes Are Uniformly Deployed in the Network

In Scenario 1, we uniformly deploy 50, 100, 200, 400, 800, and 1200 nodes in the network. The mobile sink gathers sensing data along the trajectory that follows the adjustable trajectory based on node density in specific subareas, the trajectory based on network size, and the trajectory based on node density of the whole network. Then the packet delivery ratio is evaluated according to the simulation results. The orders of the Hilbert curves selected by each trajectory according to different number of nodes are shown in Table 3.

It can be seen that when the number of nodes is set to 400 or larger, our ad-based curve achieved a 23.43%–29.70% improvement in PDR over the k-based curve and a 1.03%–4.21% improvement in PDR over the d-based curve, respectively, in uniform node deployment.

**Figure 8:**
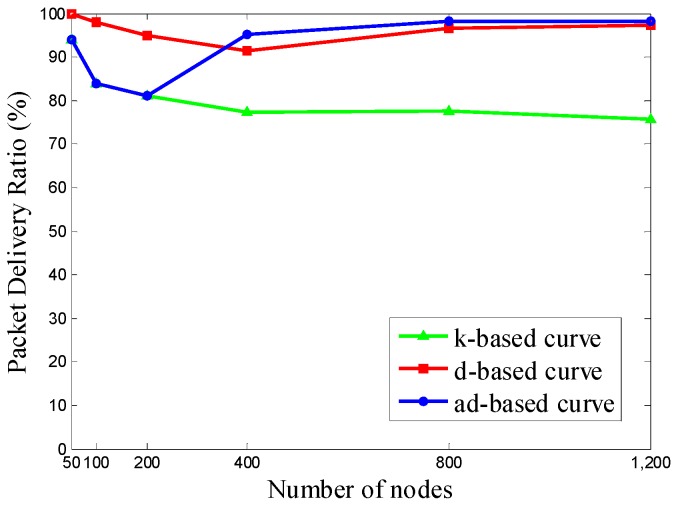
The PDR of the k-based, d-based and adjustable d-based trajectories.

As shown in Figure 8, when the number of nodes is less than 400, the PDR under the d-based curve outperforms the others, since it begins with a 2-order Hilbert curve, while the other two trajectories start from a 1-order Hilbert curve. When the number of nodes rises to 400, the PDR under the adjustable d-based trajectory outperforms the others and remains the best as the number of nodes increases to 1200.

There is no doubt that a higher order Hilbert curve can achieve a better PDR in densely deployed networks. The d-based and the adjustable d-based curves use higher curve orders than that in a k-based curve according to the node density, which is the main reason that the PDR under these two curves can outperform the k-based curve. The reason that the adjustable d-based curve can surpass the d-based curve is that the adjustable d-based curve uses adaptive power control to adjust the transmission range of sensor nodes, and thus it relieves the communication congestion and achieves the desired PDR.

**Scenario 2.** Sensor Nodes Are Unevenly Deployed in the Network

**Figure 9:**
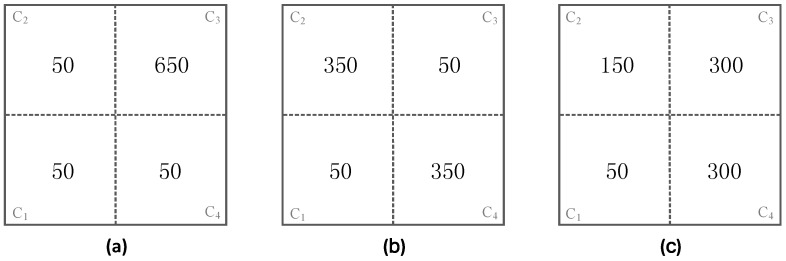
Three kinds of node deployments in Scenario 2: (**a**) C_1_, C_2_, C_4_ are deployed with 50 nodes and C_3_ is deployed with 650 nodes; (**b**) C_1_, C_3_ are deployed with 50 nodes and C_2_, C_4_ are deployed with 350 nodes; (**c**) C_1_ is deployed with 50 nodes, C_2_ is deployed with 150 nodes, C_3_ and C_4_ are all deployed with 300 nodes.

**Figure 10:**
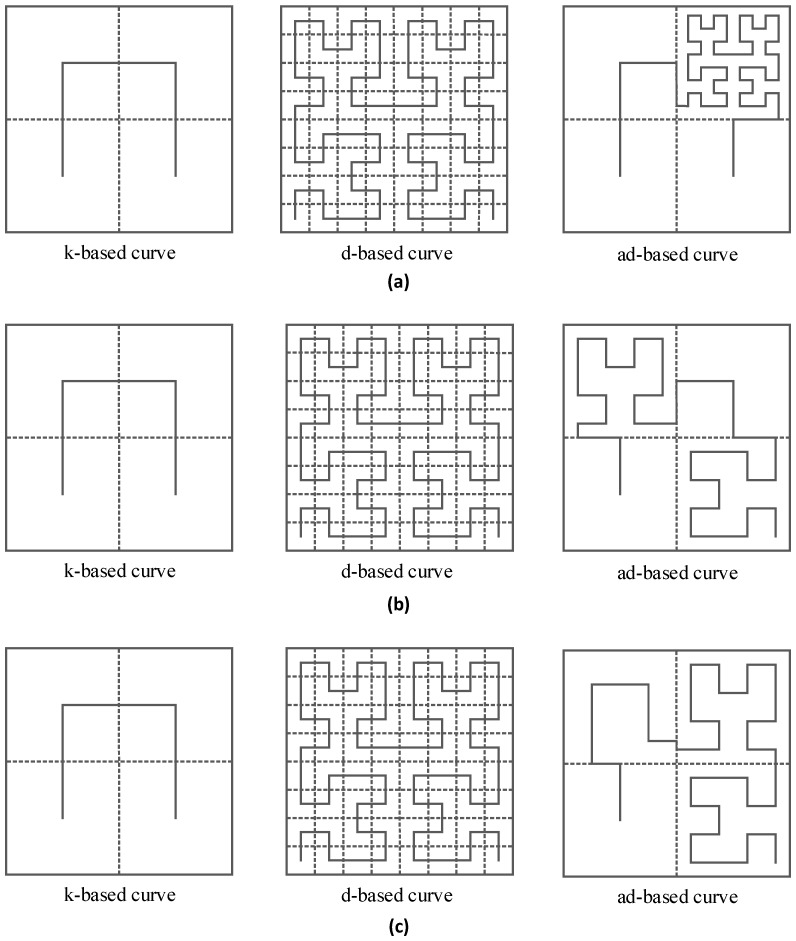
Illustration of k-based, d-based and adjustable d-based trajectories in Scenario 2: (**a**) the three kinds of trajectories in Figure 9a; (**b**) the three kinds of trajectories in Figure 9b; and (**c**) the three kinds of trajectories in Figure 9c.

In Scenario 2, to verify our proposed adjustable trajectory based on node density in specific subareas, sensor nodes are unevenly deployed, whereby some subareas are densely deployed, and the other subareas are sparsely deployed. The number of nodes in the network is set to 800 and there are three kinds of node deployments adopted in this scenario, which are shown in Figure 9a–c, respectively. Figure 10 shows the corresponding k-based, d-based and adjustable d-based trajectories in each kind of node deployment in this scenario, respectively.

In Figure 9a,b, only one or two subareas are densely deployed while the other subareas are sparsely deployed. In Figure 9c, to further verify the flexibility of these three kinds of trajectories, the node densities in C_1_, C_2_, C_3_ are different from each other and C_3_, C_4_ are densely deployed.

In Figure 9a, it can be seen that nodes in subarea C_3_ are densely deployed while in the other three subareas they are sparsely deployed. Since the network is divided into four subareas and C_3_ includes 650/800 = 81.25% nodes of the whole network, the PDR in C_3_ contributes most to the overall PDR. Similarly, in Figure 9b,c, the PDR in subareas with the largest node density can affect the overall PDR the most.

**Figure 11:**
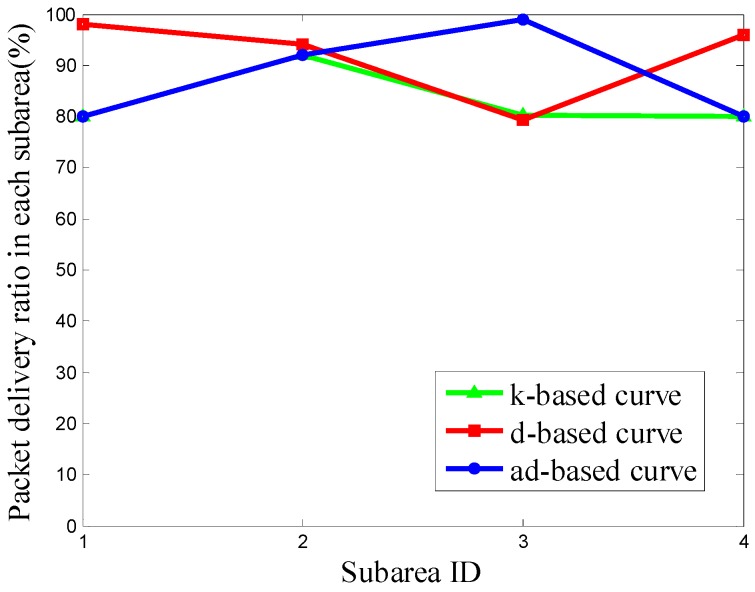
The PDR of k-based, d-based and adjustable d-based curve in node deployment (a) in Scenario 2.

**Figure 12:**
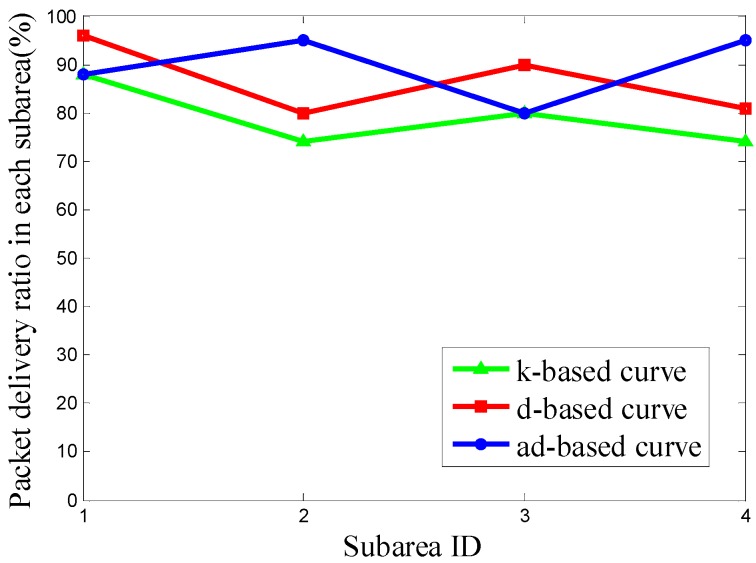
The PDR of k-based, d-based and adjustable d-based curve in node deployment (b) in Scenario 2.

**Figure 13:**
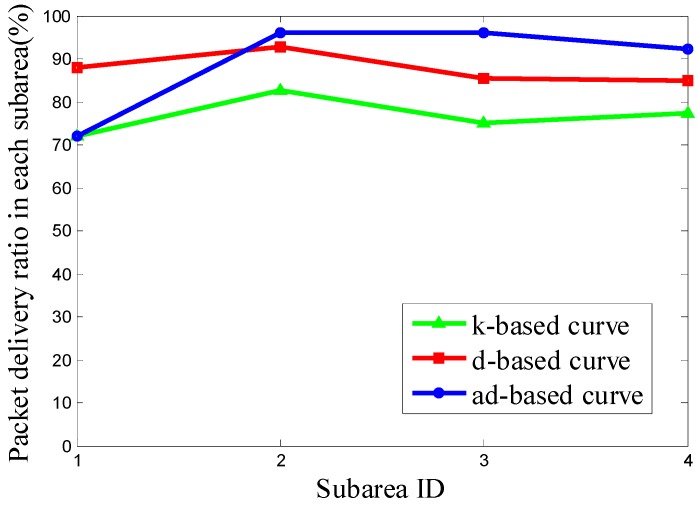
The PDR of k-based, d-based and adjustable d-based curve in node deployment (c) in Scenario 2.

It can be seen from Figure 11 that the PDR using the d-based curve outperforms the others in subareas C_1_, C_2_ and C_4_. However, in subarea C_3_, where most nodes are deployed, the adjustable d-based curve gets much better PDR than the other two curves. Similarly, in Figure 12 and Figure 13, the PDR under the ad-based curve outperforms that under the other two curves in subareas with dense node deployment. Therefore, as shown in Table 4, the adjustable d-based curve achieves the highest overall PDR among these three kinds of trajectories in all three kinds of node deployments. The reason is that the adjustable d-based curve adopts the proper order of the Hilbert curve to adapt to the highly dense deployment subarea, while the other two curves do not. The adaptive power control in an adjustable trajectory based on node density helps relieve the network traffic congestion, thus the adjustable d-based curve can achieve the desired PDR. From the simulation results and detailed analysis above, the ad-based curve outperforms the k-based curve and d-based curve by 21.20% and 12.76% on average in packet delivery ratio in Scenario 2, respectively.

### 5.2. Evaluation on Network Lifetime in a Single-Hop Model

**Scenario 3.** Sensor Nodes are Uniformly Deployed in the Network

**Figure 14:**
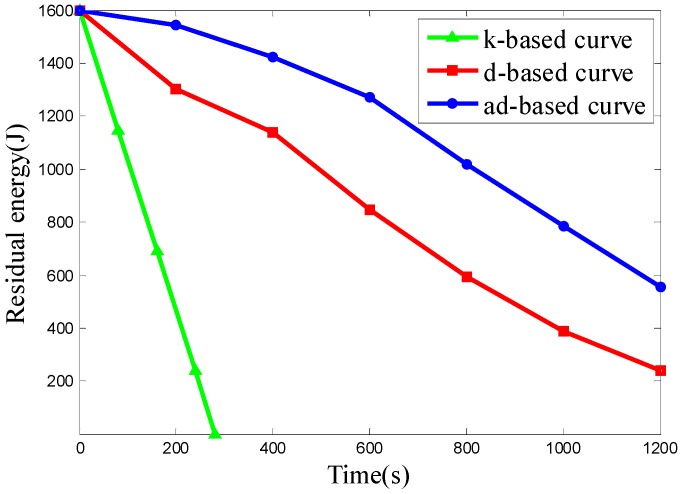
The residual energy of the network in Scenario 3.

In Scenario 3, the number of nodes is set to 800 and the nodes are deployed uniformly in network. The k-based, d-based and adjustable d-based curves are evaluated in energy consumption. Figure 14 shows the residual energy of the network under these three kinds of trajectories by time.

From the simulation results above, it can be seen that the ad-based curve achieves much better results on residual energy than the k-based curve and consumes 23.09% less energy than the d-based curve in uniform node deployment.

Since all the 800 nodes are deployed uniformly in the network, both the d-based curve and adjustable d-based curve use a 3-order Hilbert curve, while the k-based curve uses a 1-order Hilbert curve. It can be seen from Figure 14 that the energy consumption using adjustable d-based curves is much less than that with the k-based curve and d-based curve, which makes the network save more energy.

The k-based curve drains the battery much faster than the other two curves due to the high packet transmission rate, while using the other two curves can reduce the packet transmission rate by using a higher order Hilbert curve, thus reducing the energy consumption. The energy consumption with an ad-based curve is even less than that with a d-based curve. In an adjustable d-based curve, the transmission ranges of the sensor nodes to communicate with the mobile sink are reduced by the adaptable power control, which is the main reason that the adjustable d-based curve achieves better performance than d-based curves in energy consumption.

**Scenario 4.** Sensor Nodes Are Unevenly Deployed in the Network

**Figure 15:**
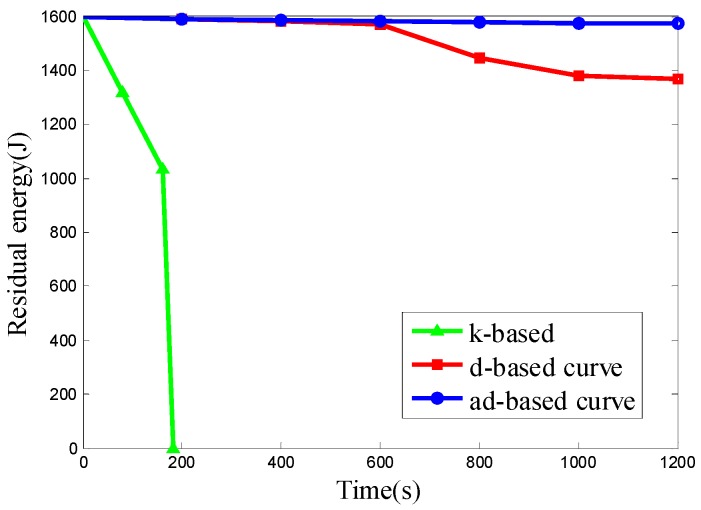
The residual energy of the network in node deployment (a): C_1_, C_2_, C_4_ are deployed with 50 nodes and C_3_ is deployed with 650 nodes.

**Figure 16:**
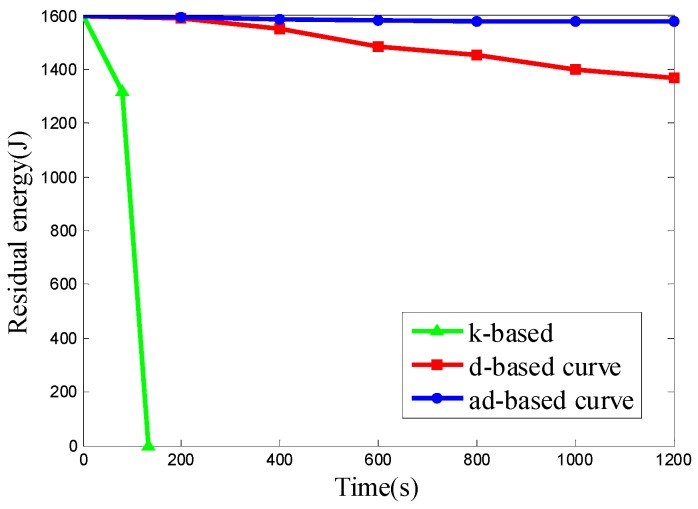
The residual energy of the network in node deployment (b): C_1_, C_3_ are deployed with 50 nodes and C_2_, C_4_ are deployed with 350 nodes.

**Figure 17:**
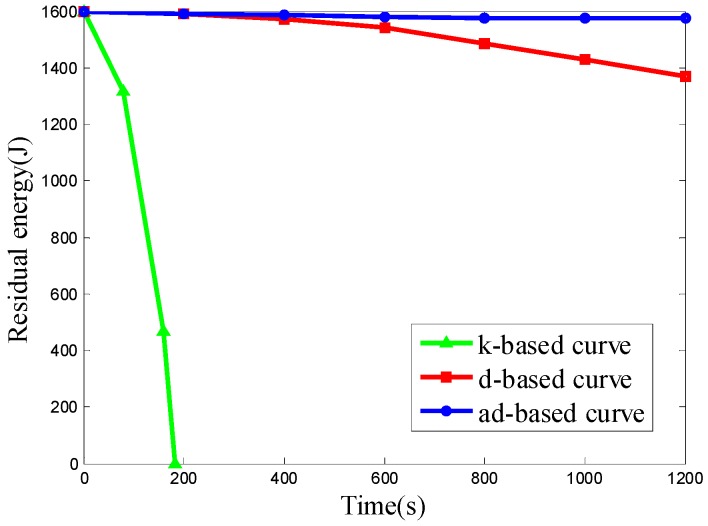
The residual energy of the network in node deployment (c): C_1_ is deployed with 50 nodes, C_2_ is deployed with 150 nodes, C_3_ and C_4_ are deployed with 300 nodes.

We want to evaluate the energy consumption of the k-based, d-based and adjustable d-based curves in a scenario with different node densities in specific subareas. Therefore the node deployments used in Scenario 4 are the same as those in Scenario 2. The experimental results of the three kinds of node deployment are shown in Figure 15, Figure 16 and Figure 17, respectively.

It can be seen from the results above that the ad-based curve consumed 89.02%, 89.02% and 89.12% less energy, respectively, than the d-based curve in the three kinds of uneven node deployment. It is observed that the energy consumption under the k-based curve scenario is much faster than that under the d-based and ad-based curves. By using the adaptable power control scheme and selecting the proper curve order in each subarea, the average energy consumption with the adjustable d-based curve is less than that with the d-based curve. In the simulation, when the mobile sink traverses to a subarea with high node density, the k-based curve continues to use a 1-order Hilbert curve, thus resulting in a high transmission rate and high energy consumption. Similarly, the d-based curve uses a d-order Hilbert curve that is based on the node density of the whole network, so compared with the adjustable d-based curve, it is still not flexible enough to reflect the real node deployment in a specific subarea, while in an adjustable d-based curve, the proper curve order is used to increase the PDR according to the node density in specific subareas and the transmission ranges of the nodes are reduced according to the curve order in this subarea, therefore the ad-based curve achieves the best energy efficiency among these three kinds of trajectories, and we can conclude that it is better to use an ad-based trajectory than a k-based or d-based trajectory in such a network with uneven node deployment.

### 5.3. Evaluation on Packet Delivery Ratio in a Cluster Model

**Scenario** **5.** Sensor Nodes Are Unevenly Deployed in the Network

**Figure 18:**
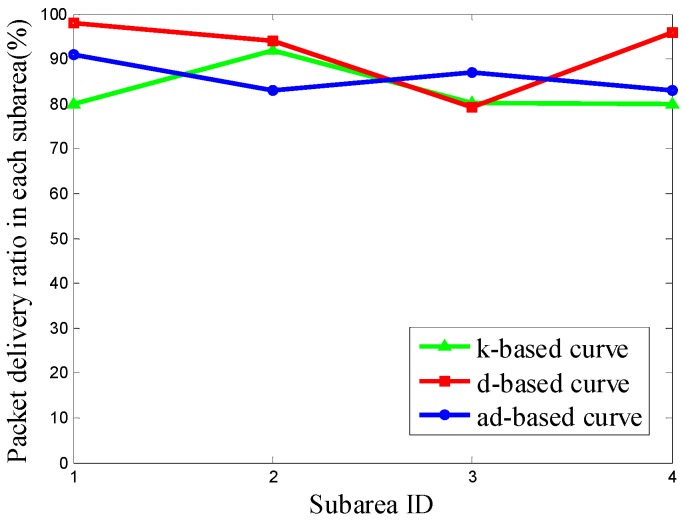
The PDR of k-based, d-based and adjustable d-based curve in node deployment (a): C_1_, C_2_, C_4_ are deployed with 50 nodes and C_3_ is deployed with 650 nodes.

**Figure 19:**
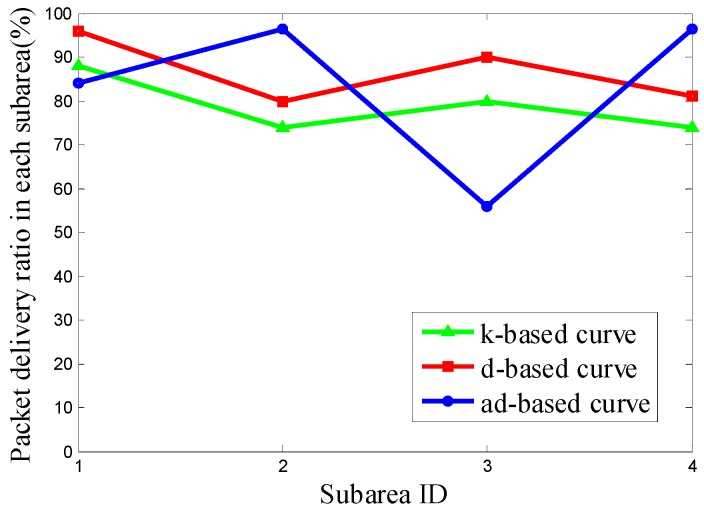
The PDR of k-based, d-based and adjustable d-based curve in node deployment (b): C_1_, C_3_ are deployed with 50 nodes and C_2_, C_4_ are deployed with 350 nodes.

**Figure 20:**
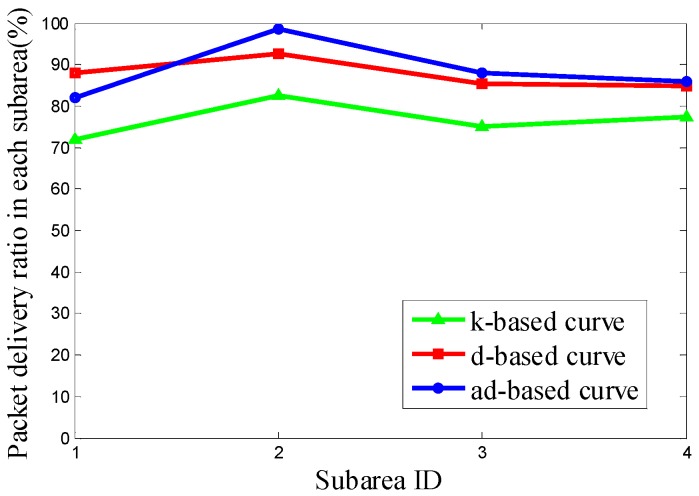
The PDR of k-based, d-based and adjustable d-based curve in node deployment (c): C_1_ is deployed with 50 nodes, C_2_ is deployed with 150 nodes, C_3_ and C_4_ are deployed with 300 nodes.

In this scenario, the number of nodes is set to 800 and the sensor nodes are deployed using the three kinds of node deployments in Scenario 2. Then the PDR for all the three kinds of trajectories which are proposed: ad-based curve in a cluster model, k-based curve and d-based curve are evaluated. The results in these three kinds of node deployments are shown in Figure 18, Figure 19 and Figure 20, respectively.

Table 5 gives the overall PDR of these three trajectories in this scenario.

It can be seen from Table 5 that the ad-based curve in the cluster model achieves the desired overall PDR and on average it outperforms the k-based and d-based curves in PDR by 11.40% and 3.64%, respectively, in this scenario.

In a subarea with sparse node deployment, the CHs have to collect all the sensing data in their clusters first, and then send is to the mobile sink, which also means that each CH has to send more sensing data to the mobile sink than a sensor node in the single-hop model, where the sensor nodes can send the sensing data to the mobile sink directly. However, since the time the mobile sink stays in one subarea is the same as that in the single-hop model, such a time period may not be enough for the mobile sink to collect all the sensing data from all CHs, which in return results in the decrease in PDR for this subarea. In a subarea with dense node deployment, the number of sensor nodes in one cluster increases due to the higher node density, thus the CHs have to send even more data to the mobile sink. However, the ad-based curve can dynamically select a higher order Hilbert curve, which results in an increase of staying time of the mobile sink in this subarea, and thus there may be enough time for the mobile sink to fully collect all the sensing data from all the CHs, therefore, it can result in the increase in PDR for this subarea.

By comparing the overall PDR of the ad-based curve with different communication models, which are the single-hop model in Scenario 2 and the cluster model in this scenario, it can be seen that the ad-based curve in a single-hop model outperforms that in a cluster model in PDR by 9.69% on average.

For a cluster model, in the first place, this kind of two-step communication has effects on performance features such as latency, each CH (relay node) incurs additional delays, e.g., for queuing the data. On the other hand, communications over a two-step model may often be less reliable, enhancing the communication time and overhead, since the same data must be transmitted several times (and the limited channel capacity must be shared among the relays for both receiving and transmitting), so the maximum achievable throughput will also suffer. Further, reliability can be affected by having multiple nodes (relays) involved in the communication which increases the risk of disruptions due to link or node failures. Therefore, the PDR for ad-based trajectory in a cluster model is less than that in a single-hop model.

### 5.4. Evaluation on Network Lifetime in a Cluster Model

**Scenario** **6.** Sensor Nodes Are Unevenly Deployed in the Network

**Figure 21:**
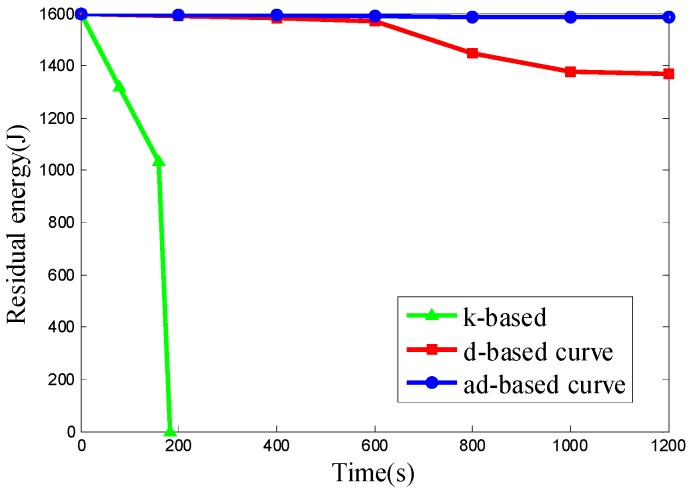
The residual energy of the network in node deployment (a): C_1_, C_2_, C_4_ are deployed with 50 nodes and C_3_ is deployed with 650 nodes.

**Figure 22:**
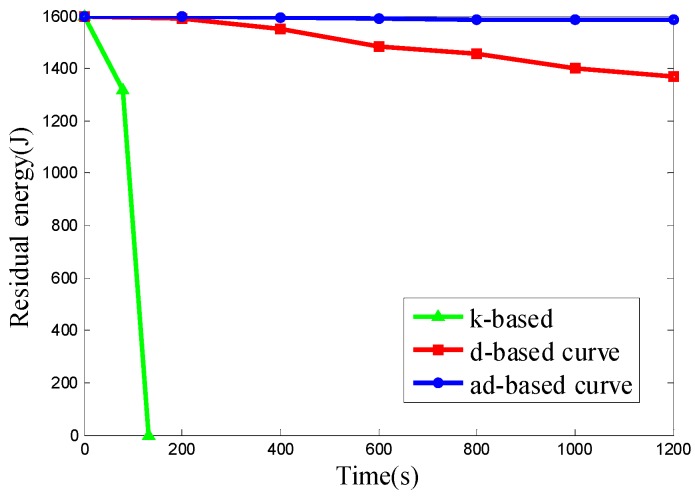
The residual energy of the network in node deployment (b): C_1_, C_3_ are deployed with 50 nodes and C_2_, C_4_ are deployed with 350 nodes.

**Figure 23:**
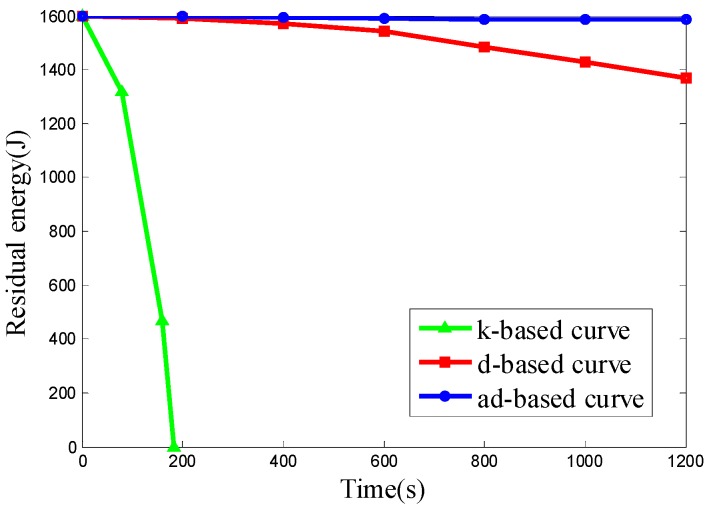
The residual energy of the network in node deployment (c): C_1_ is deployed with 50 nodes, C_2_ is deployed with 150 nodes, C_3_ and C_4_ are deployed with 300 nodes.

In this scenario, the number of nodes is set to 800, and nodes are also deployed using the three kinds of node deployments used in Scenario 2. The simulation results are shown in Figure 21, Figure 22 and Figure 23, respectively.

It can be seen from the above results that the ad-based curve in the cluster model consumed 94.82%, 94.06% and 94.05% less energy than the d-based curve in the three kinds of uneven node deployment, respectively.

The simulation results show that in Scenario 6, the k-based curve is also the first one to drain its battery. There is no doubt that the high retransmission rate in the k-based curve case results in high energy consumption. By using the cluster model and the adaptive power control, the transmission power of the sensor nodes is minimized while the transmission power of CHs is adjusted according to the curve order of the trajectory in the ad-based curve in the cluster model. Therefore, the ad-based curve in the cluster model achieves the best energy efficiency among these three trajectories.

By comparing the energy consumption for the ad-based curve in the single-hop model in Scenario 4 and the ad-based curve in the cluster model in this scenario, it can be calculated that the ad-based curve in the cluster model consumed 52.85%, 41.08% and 45.35% less energy than that in single-hop model in the three kinds of node deployment, respectively.

By using the cluster model, the data gathering process of the ad-based curve consists of two steps. In the first step, most of the sensor nodes only need to transmit their sensing data to the CHs which are close to them other rather than the mobile sink that is far from them. Therefore, the reduction in transmission distance can ultimately result in an energy consumption decrease based on our energy model. In the second step, the CHs can send the gathered sensing data to the mobile sink using transmission power adjusted by the adaptive power control method, however, due to the increase of the data size and communication distance, the energy consumption for CHs is higher than that in the single-hop model, but it can be known that, in the cluster model, most of the sensor nodes only need to communicate with the CHs over a small distance while only a few nodes (which are CHs) need to communicate with the mobile sink over a long distance, and therefore, the overall energy consumption for the whole network can be reduced dramatically.

From the above work, we can draw the conclusion that, the ad-based curve in single-hop communication model has the highest PDR and the ad-based curve in the cluster model has the highest energy efficiency. Therefore, the communication model can be properly selected to maximize the PDR or the network lifetime, according to the application requirement.

## 6. Conclusions

In this paper, an adjustable trajectory design method is developed by using the Hilbert space-filling curve, in which the full coverage of the network and the desired packet delivery ratio are achieved. By selecting the proper order of the Hilbert curve based on the node density in specific subareas, more sensing data can be gathered and the network performance is improved in densely deployed networks with uneven node deployment. In addition, the proposed trajectory also saves energy and prolongs the network lifetime. Simulation results demonstrate that the proposed trajectory is flexible and can adapt to networks with different node density or node deployment. It is also verified that the proposed trajectory outperforms the existing classic trajectories in the packet delivery ratio and the network energy efficiency with the designed adaptable power control scheme.

One future project is how to design a more flexible method of network partition, so the sensor nodes in a subarea can work cooperatively to collect the sensing data using a smaller transmission range, and we can consider using more sophisticated routing mechanisms, such as opportunistic routing, to deliver the sensing data to the mobile sink, in order to decrease the average packet delay in each round of data gathering, which is very important in event-driven WSNs. Another future direction of this work is how to use multiple mobile sinks to increase the data gathering efficiency, and hoe to apply an energy rechargeable algorithm and energy consumption model considering the energy consumptions of both transmission and computing [24,25,26].

## Figures and Tables

**Table 1:** The format of the power control message.

Message Type	Target Subarea ID	New TX Range

**Table 2:** Simulation parameters.

Parameters	Values
Network Size	800 × 800 m^2^
Number of Nodes in Network	50, 100, 200, 400, 800, 1200
Number of Mobile Sink	1
Movement Speed of Mobile Sink	5 m/s
Mobility Model	Hilbert space-filling curve
Sensing Range	100 m
MAC	802.11
Initial Energy (E)	2 J
Transmission Power (P_tx_)	0.05–2.6 mW
Reception Power (P_rx_)	1 mW

**Table 3:** The curve order of k-based, d-based and adjustable d based curve for different number of nodes.

No	Number of Nodes	k-Based Curve Order	d-Based Curve Order	Adjustable d-Based Curve Order
1	50	1	2	1 (in all subareas)
2	100	1	2	1 (in all subareas)
3	200	1	2	1 (in all subareas)
4	400	1	2	2 (in all subareas)
5	800	1	3	3 (in all subareas)
6	1200	1	3	3 (in all subareas)

**Table 4:** The overall PDR of k-based, d-based and adjustable d-based curves in Scenario 2.

Node Deployment (a) In Scenario 2		Node Deployment (b) In Scenario 2		Node Deployment (c) In Scenario 2	
Trajectory	Overall PDR	Trajectory	Overall PDR	Trajectory	Overall PDR
k-based curve	80.88%	k-based curve	75.38%	k-based curve	77.13%
d-based curve	82.38%	d-based curve	81.75%	d-based curve	86.75%
ad-based curve	96.25%	ad-based curve	93.50%	ad-based curve	93.13%

**Table 5:** The Overall PDR of k-based, d-based and adjustable d-based curves in Scenario 5.

Node Deployment (a) In Scenario 5		Node Deployment (b) In Scenario 5		Node Deployment (c) In Scenario 5	
Trajectory	Overall PDR	Trajectory	Overall PDR	Trajectory	Overall PDR
k-based curve	80.88%	k-based curve	75.38%	k-based curve	77.13%
d-based curve	82.38%	d-based curve	81.75%	d-based curve	86.75%
ad-based curve	86.00%	ad-based curve	83.25%	ad-based curve	88.63%
